# Attosecond science based on high harmonic generation from gases and solids

**DOI:** 10.1038/s41467-020-16480-6

**Published:** 2020-06-02

**Authors:** Jie Li, Jian Lu, Andrew Chew, Seunghwoi Han, Jialin Li, Yi Wu, He Wang, Shambhu Ghimire, Zenghu Chang

**Affiliations:** 1 Academy of Opto-Electronics, Chinese Academy of Sciences, Beijing, 100094 China; 2 Institute for the Frontier of Attosecond Science and Technology, CREOL and Department of Physics, University of Central Florida, Orlando, FL 32816 USA; 3 School of Optoelectronics, University of the Chinese Academy of Sciences, Beijing, 100049 China; 4 Stanford PULSE Institute, SLAC National Accelerator Laboratory, Menlo Park, CA 94025 USA; 5 School of Mechanical Engineering, Chonnam National University, Gwangju, 61186 Republic of Korea; 6 Department of Physics, University of Miami, Coral Gables, FL 33146 USA

**Keywords:** High-harmonic generation, Attosecond science

## Abstract

Recent progress in high power ultrafast short-wave and mid-wave infrared lasers has enabled gas-phase high harmonic generation (HHG) in the water window and beyond, as well as the demonstration of HHG in condensed matter. In this Perspective, we discuss the recent advancements and future trends in generating and characterizing soft X-ray pulses from gas-phase HHG and extreme ultraviolet (XUV) pulses from solid-state HHG. Then, we discuss their current and potential usage in time-resolved study of electron and nuclear dynamics in atomic, molecular and condensed matters.

## Abstract (web summary)

Different methods are demonstrated in recent years to produce attosecond pulses. Here, the authors discuss recent development and future prospects of the generation of such pulses from gases and solids and their potential applications in spectroscopy and ultrafast dynamics in atoms, molecules and other complex systems.

## Introduction

Tabletop attosecond light sources in the soft X-ray (SXR) spectral region based on high-harmonic generation are highly desirable in chemical and material sciences since they can spectroscopically identify specific elements, as well as the oxidation states, charge states and even the spin states of those elements^1^. One of the important spectral regions is the “water window” (282–533 eV), which covers the atomic K-shell excitation of carbon and oxygen. Although high harmonics in the water window were first generated with Ti:Sapphire lasers centered at 800 nm more than 20 years ago^2,3^, the X-ray photon flux was too low for time-resolved applications.

The mechanism of HHG in gases can be explained by the semiclassical three-step model^4–6^. When driving laser-field strength reaches ~10^8^ V m^−1^, the bound electron in the atomic gas can tunnel through the Coulomb potential barrier and become a free electron. In the oscillating laser field, the free-electron wave packet may return to its parent ion with the right time of birth. At recombination, the interference between the wave packets of the returning and bound electrons produces an oscillating dipole that emits attosecond radiation. Returning electrons with various kinetic energy will recombine at different times giving rise to the chirp in the attosecond radiation^7^. This process repeats twice for every optical cycle. The temporal beating of attosecond pulses results in the high-harmonic combs in the spectral domain.

Empowered by the advances in driving lasers with center wavelengths around 1.8 μm, soft X-ray high harmonics can be generated with a moderate intensity of 10^14^ W cm^−2^ (see Box 1 for details). Significant progress has recently been made in developing attosecond light within the water window^8^. By spectrally broadening pulses from an Optical Parametric Amplifier (OPA) using a gas-filled hollow-core fiber^9^ or by broadband phase matching in an Optical Parametric Chirped Pulse Amplifier (OPCPA)^10^, two-cycle, mJ-level pulses centered with 1 kHz repetition rate are now generated in many laboratories^8^. Seed pulses for such light source can be produced by intra-pulse difference frequency generation (DFG)^11^ with stable carrier–envelope phase.

In 2010 HHG has also been realized in condensed matter systems using mid-infrared laser fields^12^. Since then, there has been significant attention on this field in investigating the underlying microscopic mechanism of solid-state HHG. Its promises include stable attosecond light sources in compact forms and the possibility of attosecond metrology in solid-state materials^13,14^. A string of experimental surprises in solid-state HHG include anomalous ellipticity dependence^15^, observation of a multiple plateau feature^16^ and orthogonally polarized high harmonics^17^. XUV harmonics from certain solid materials such as SiO_2_ crystals were found to be immune to fluctuations in the driving pump laser, providing a novel path toward stable attosecond pulses^18^. There are many exciting initiatives involving the exploitation of microscopic generation processes in high-harmonic spectroscopy (HHS) of materials, including all-optical probing of the valence charge density in the real space^15,19^ and electronic band structure in the reciprocal space^20^. Other unique features include the use of engineered materials at the nanometer scale to enhance, control, and manipulate the generation process. HHS provides a novel approach to probe active electrons on nanoscale devices and on the surface state of quantum materials such as topological insulators.

In this perspective, we will first discuss the recent progress, challenges, and future trends in the development of gas-phase attosecond X-ray sources in the water window, followed by an extensive discussion on their applications in molecular systems and condensed matter. Then, we will briefly review novel microscopic dynamics underlying solid-state HHG and discuss their applications in high-harmonic spectroscopy of condensed matter systems both in and out-of-equilibrium.

Box 1 Photon energy of the high-harmonic spectrum The highest photon energy (cutoff photon energy) from gas-phase HHG can be estimated by

1 $$\hbar \omega _{\mathrm{c}}\left[ {eV} \right] = I_{\mathrm{p}} + 3 \times 10^{ - 13}I_0\lambda _{\mathrm{L}}^2,$$

where *I*_p_ is the ionization potential of the gas atom expressed in eV. The peak laser intensity $I_0$ is in W cm^−2^, and the driving laser wavelength, $\lambda _{\mathrm{L}}$, is in μm. Due to quantum diffusion, the single-atom efficiency decreases with the driving laser wavelength (~$\lambda _{\mathrm{L}}^{ - 6}$)^41^. The peak laser intensity is set for phase matching the HHG process to achieve high photon flux, where the negative plasma dispersion is balanced by the unionized portion of the target gas. The calculated cutoff photon energy for noble gases at different laser wavelengths is depicted in the figure below. This shows that the HHG spectrum can cover the water window by using short-wave infrared (SWIR) lasers^144,145^. Ionization induced plasma defocusing tends to clamp laser intensity and reshape the radius profile^146,147^. The achievable SXR spectrum is therefore a collaborative result of atomic response and coherent buildup of high-harmonic strength.

**Wavelength scaling of HHG cutoff photon energy**. Calculated cutoff photon energy of HHG under the phase-matching condition from various inert gases driven by lasers at different center wavelengths. The purple square, blue dot, and red triangle indicate the achievable cutoff photon energy by using a 0.8 μm, 1.6 μm, and 2.5 μm driving laser, respectively.

## Isolated water window X-ray attosecond pulses

Single isolated attosecond pulses (IAP) are needed for conducting pump–probe measurements, such as attosecond streaking and attosecond transient-absorption spectroscopy^21–24^. Various gating techniques have been developed to obtain such pulses (Table 1), and some of them have been implemented to generate isolated water window X-ray bursts.

**Table 1:** Overview of gas-phase isolated attosecond light sources.

Gating method (gas)	Central energy/ FWHM (eV)	Pulse duration/FT limit (as)	Pulse energy	Peak power (MW)	Characterization	Year/refs.
PG (Ne)	100/22	130/45	70 pJ	0.5	FROG-CRAB	2006/CNR-IFN/Italy^136^
AG (Ne)	83/28	80/75	0.5 nJ	6	FROG-CRAB	2008/MPQ/Germany^137^
IG (Xe)	25/8	155/130	9 nJ	60	FROG-CRAB	2010/CNR-IFN/Italy^138^
DOG (Ne)	100/40	67/16	N/A	N/A	PROOF	2012/UCF/USA^139^
Lighthouse (Ne)	50/35	(N/A)/47	N/A	N/A	N/A	2013/NRC/Canada^140^
TC (Xe)	30/7	500/(N/A)	1.3 μJ	2600	Autocorrelation	2013/RIKEN/Japan^141^
PG (Ne)	170/120	53/20	3 pJ	0.05	PROOF	2017/UCF/USA^36^
AG (Ne)	110/80	43/34	N/A	N/A	ML-VTGPA	2017/ETHZ/Switzerland^142^
AG (Ne)	250/100	<322/20	40 pJ	0.1	FROG-CRAB	2017/ICFO/Spain^26,143^
DOG (Ne)	150/100	305/(N/A)	0.3 nJ	1	Neural network	2019/UCF/USA^40^

*FWHM* full-width at half-maximum, *FT* Fourier transform.

### Subcycle gating techniques

The simplest method to isolate a single attosecond burst from a few-cycle driving field is the amplitude gating (AG)^25^. In AG, with a proper carrier–envelope phase value, the strongest half-cycle of the driving field will produce the attosecond burst with the broadest spectrum that exceeds all other bursts. By selecting a suitable filter that transmits only this cutoff spectrum, an IAP can be achieved. AG has been adapted for the few-cycle short-wave infrared laser to demonstrate water window IAP^10,26–28^. The width of the continuum near the cutoff can be estimated by

2 $${\mathrm{\Delta }}\hbar \omega _{\mathrm{c}}[eV] = 3 \times 10^{ - 13}{\mathrm{\Delta }}I_{\mathrm{L}}\lambda _{\mathrm{L}}^2,$$

where ${\mathrm{\Delta }}I_{\mathrm{L}}$ is the intensity difference between the most intense half-cycle and the neighboring half-cycles of the driving laser field. Future efforts will be made to increase this intensity by reducing the SWIR driving laser-pulse duration from the current state-of-the-art, ~two cycle, to just a single cycle. It is clear from Eq. (2) that a much broader continuum can be obtained by using a longer wavelength driving laser for a given intensity difference. Highly efficient Chirped Pulse Amplification lasers based on gain media such as Cr:ZnSe at 2.5 μm and Fe:ZnSe at 4 μm may extend the attosecond spectrum to O K-edge^29–31^.

Spectral filtering methods, such as AG and ionization gating (IG)^32^, can only isolate IAPs near the HHG spectrum cutoff. Techniques based on the temporal gating of the HHG process can generate an attosecond supercontinuum that covers both the plateau and cutoff spectrum portion. The most common temporal gating method is polarization gating (PG)^33,34^. The influence of field ellipticity on HHG suppression increases as the driving wavelength increases^35^. PG has the potential for generating ultra-broadband SXR pulses^36^, which may support even shorter attosecond sources in the future. Unfortunately, in PG, a substantial amount of laser energy is wasted^37^. To reduce loss, the double optical gating (DOG) technique was proposed^38^. In DOG, a second harmonic field is added to break the fundamental field’s symmetry in a process known as two-color gating^39^. A stronger IAP in the water window can be expected by applying the DOG^40^.

### Characterization of attosecond pulses

Due to the low conversion efficiency (~10^−6^) in gas-phase^41^ high-harmonic generation, attosecond pulse metrology relies on the photon ionization of the gas media by a weak IAP in the presence of a perturbative infrared (IR) pulse. The photoelectron will gain (lose) energy by absorbing (emitting) one or more IR photons, depending on their relative delay. This technique, named attosecond streaking^24^, can characterize an IAP after its birth. The unknown attosecond spectral phase information is encoded in the perturbed photoelectron energy spectrogram and can now be retrieved using the frequency-resolved optical gating for complete reconstruction of attosecond bursts (FROG-CRAB) technique^42^, which is suitable for narrowband pulses. Alternative techniques are available for characterizing broadband pulses^43–46^.

Statistical noise in the streaking traces pose a challenge to reliable phase retrieval. For instance, large discrepancies are found in the pulse duration and spectral phase when different phase retrieval schemes are applied^47^. Previous attosecond phase-retrieval schemes are based on time-consuming iterative algorithms. It is expected that deep neuron network (DNN) algorithms will significantly cut down retrieval time^46^. Streaking measurements are affected by space charge effects. SWIR OPA/OPCPA lasers based on high-average-power diode-pumped thin disc/slab Yb lasers^48^ provide a promising approach to deliver high-repetition-rate and high-flux attosecond pulses in the water window. This will significantly improve the signal to noise ratio of streaking traces.

### Atto-chirp compensation

In HHG experiments, usually the short quantum pass survives through the macroscopic phase matching resulting in attosecond pulses with a positive chirp^49^. Such chirp can be compensated by the negative group delay dispersion (GDD) of thin foils or neutral gases^50^. Recently, isolated 53-as X-ray pulses whose spectra cover the 100–300 eV range have been characterized by attosecond streaking^36^. It is, however, difficult to compensate atto-chirp above the carbon K-edge (282 eV) due to the lack of materials that exhibiting negative group velocity dispersion and low loss. It was illustrated theoretically that atto-chirp in the 300–1000 eV range can be reduced by hydrogen gas or plasma with the proper pressure–length product given that the transmission of the gas or plasma is higher than 10%^51,52^.

**Fig. 1:**
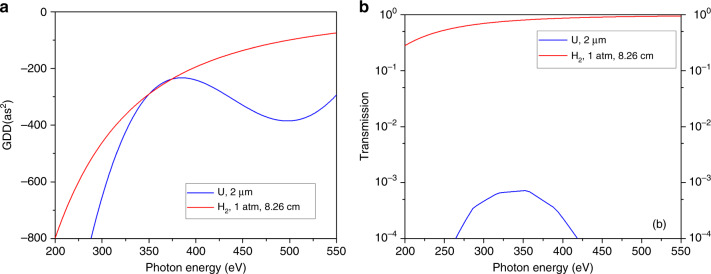
Atto-chirp compensation in the water window. Comparison of group delay dispersion (**a**) and X-ray transmission (**b**) between a 2-μm-thick uranium foil and 8.26 cm long, 1 atm H_2_ gas from 250 to 550 eV.

The GDD of neutral molecular hydrogen gas at the photon energy $\hbar \omega _{\mathrm{x}}$ can be expressed

3 $$GDD_{{\mathrm{H}}_2}\left( {\hbar \omega _{\mathrm{x}}} \right) = 1.509 \times 10^9\frac{{PL}}{{\left( {\hbar \omega _{\mathrm{x}}} \right)^3}},$$

where pressure, *P*, is in atm, length, *L,* is in cm, and photon energy, ℏ*ω*_x_,  is in eV. As an example, to compensate a 1600 as^2^ chirp at 365 eV, the required pressure–length product is 54-atm cm, which is experimentally feasible. Hydrogen is preferred because of its low absorption in the SXR region compared with other materials. Uranium foil also exhibits negative GDD beyond 300 eV^53^, but its absorption is much higher than H_2_, as shown in Fig. 1. Their dispersion values are the same at 350 eV. The peak transmission of U foil is <10^−3^ to compensate a 290 as^2^ atto-chirp, where the transmission of H_2_ is 80%. The transmission of ionized H_2_ is even better.

Single-atom simulations based on the strong-field approximation^54–58^ of high-harmonic generation have been performed to demonstrate feasibility of the atto-chirp compensation in the 300–500 eV^51^ and 530–1000 eV^52^ photon energy regions. It has been shown that isolated attosecond pulses as short as 25-as are achievable when high-harmonic generation in helium is driven by single-cycle lasers centered at 1.7 μm or 3.2 μm. The effectiveness of the chirp compensation by H_2_ plasma has been validated by simulations that account for phase macroscopic effects^59^. Experimental demonstration of such a chirp compensation scheme may yield milestone results. Efforts need to be made to find schemes that can compensate the third-order phase error.

Atto-chirp can also be reduced in the HHG process by reshaping the driving field waveform using a multicolor synthesizer with controlled CEP and delay. Simulation has shown that nearly 60 pulses can be achieved in the 300–500 eV energy range without atto-chirp compensation^53^. This technique is also promising for atto-chirp suppression in keV range.

## Applications of attosecond X-ray sources

**Fig. 2:**
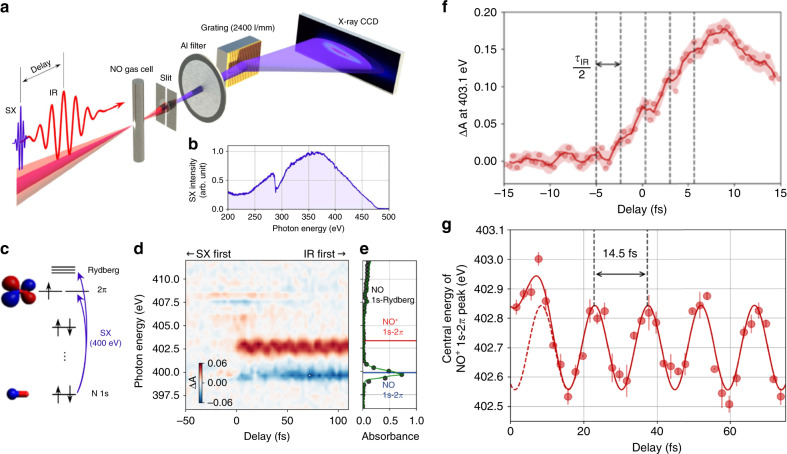
Attosecond transient-absorption spectroscopy at the N K-edge. **a** Experimental setup. **b** Attosecond X-ray spectrum. **c** Molecular orbitals of NO. **d** Absorption spectrogram. **e** Static absorbance of NO without the IR pump pulse. Reproduced from ref. ^65^, Copyright 2019, Optical Society of America. **f** Electronic response Adapted from ref. ^65^ Copyright 2019, Optical Society of America. **g** Molecular vibration. Reproduced from ref. ^65^ Copyright 2019, Optical Society of America.

Attosecond XUV sources driven by Ti:Sapphire lasers have been extensively used to study dynamics of electrons with less than 150 eV binding energy^60^. Femtosecond transient-absorption experiments with HHG sources at the carbon K-edge have recently been reported, revealing molecular structural deformation processes that happen on a 50–100-fs timescale^61,62^. Attosecond water window X-ray sources have recently enabled the observation of electronic processes in Ar atom at the L-edge (~250 eV)^63^, and in TiS_2_ films at the Ti L-edge (~460 eV)^64^. Ionization, vibration, and rotation dynamics have been resolved in NO molecules using attosecond transient-absorption spectroscopy at the Nitrogen K-edge (~400 eV)^65^, as shown in Fig. 2. It is anticipated that more experiments will be conducted to study charge dynamics in molecules and materials whose absorption edges are in the water window region^66^. Here, we discuss a few important applications.

### Charge migration in molecules and wave-package dynamics at conical intersections

Comprehensive numerical simulations of electron dynamics in polyatomic molecules have predicted that when an electron is suddenly removed from one end of a chain molecule, the hole can move to the other end in a few fs, often before electron–nuclear coupling sets in refs. ^67,68^. This purely electronic ultrafast dynamic process is termed charge migration, a convention introduced in ref. ^67^, which should be distinguished from charge transfer, a slower spatial redistribution of electronic charge involving nuclear motion that leads to charge relocation permanently from a donor to an acceptor. Charge migration is the first step in the fundamental process of electron transfer, which plays a role in photochemistry, biology, and photovoltaics. Due to the coupling between the electron and nuclear motion, it may be possible to control the chemical reactivity of molecules by manipulating their electronic motion as in the subfield dubbed attochemistry^69^. Observation of charge migration requires the preparation of a coherent superposition of electronic states and experimental tools with sufficient temporal resolution to follow charge dynamics occurring on electronic timescales. This remains a grand challenge. Only a few experimental observations of charge motion in molecules have so far been reported^70,71^.

Numerical simulations predicted that charge migration occurs in many complex organic molecules^67,68,72–75^. However, almost none of them have been verified experimentally due to the lack of experimental tools. The attosecond water window X-ray sources and transient-absorption spectroscopy provide a unique opportunity to verify these predictions. They would allow probing charge migration in molecules containing C, N, and O atoms, the halogen atoms such as Cl, Br, and I, with attosecond time resolution. Moreover, they would allow experimentalists to monitor the interplay of charge migration and nuclear motion on femtosecond timescales^76^. X-ray spectroscopy is element-specific and oxidation- and charge-state sensitive^77^. Thus, SXR provide a powerful means to measure the charge states and charge migration between atoms. The individual atoms within a molecule exhibit X-ray energy shifts, which depend on either the local chemical bonding environment or the electronic coherences themselves as charges migrate around a molecule.

It is necessary to validate new experimental tools by observing charge migration in simple molecules first and then to demonstrate their unique advantages by comparing them to previous methods. Charge migration in an iodoacetylene cation has been observed in C_2_HI^+^ using high-harmonic spectroscopy^71^, where the high-harmonic XUV spectrum itself is measured as a function of pump–probe time delay after a strong ionizing pulse. High-harmonic spectroscopy is based on the predictions of the semiclassical model of HHG that relate photon energy of a harmonic peak to the time difference between the tunneling ionization (pump) and the photon emission (probe)^78^. When C_2_HI is ionized within a fraction of an optical cycle by the electric field of an intense infrared laser pulse, a coherent electronic superposition state is created that can be expressed as an orbital mixture of the HOMO and HOMO-1 ground-state orbitals of the neutral molecule.

3 $${\mathrm{\Psi }}_{{\mathrm{MIX}}}\left( {{\mathbf{r}},t} \right) = \frac{1}{{\sqrt 2 }}\left[ {\phi _{{\mathrm{HOMO}}}\left( {\mathbf{r}} \right)e^{ - iE_{{\mathrm{HOMO}}}t/\hbar } + \phi _{{\mathrm{HOMO}} - 1}\left( {\mathbf{r}} \right)e^{ - iE_{{\mathrm{HOMO}} - 1}t/\hbar }} \right].$$

**Fig. 3:**
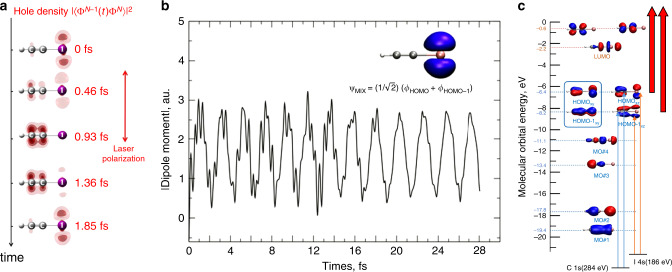
Charge migration in molecules. **a** The reconstructed electron–hole density from HHG spectroscopy as a function of time after strong-field ionization of HCCI. Reproduced from ref. ^71^ with permission from AAAS. **b** Dipole moment oscillations of C_2_HCI^+^ ionized from a HOMO/HOMO-1 superposition of states. Reproduced from ref. ^79^ (2017), AIP. **c** Charge migration measured using attosecond X-ray transient absorption. The mixed hole state in C_2_HI^+^ is formed by strong-field ionization. The valence hole is probed by core-to-valence transitions. Adapted from ref. ^79^ (2017), AIP.

Numerical simulations show that ionization from the HOMO/HOMO-1 superposition of states leads to characteristic charge oscillations with a period of ~2 fs. The dynamics of the electron hole reconstructed from the HHG measurements is shown in Fig. 3a^71^.

By definition, charge migration is periodic in the absence of dephasing or relaxation. Permanent charge transfer, on the other hand, requires electronic dephasing. One of the most important questions in the study of charge migration is the lifetime of the electronic coherence that drives charge migration especially in the presence of vibrational motion. Numerical simulations suggest that the charge migration induced by strong-field ionization lasts for at least 28 fs in C_2_HI^+^, as shown in Fig. 3b^79^. Such a prediction has not been confirmed by high-harmonic spectroscopy measurements due to the limited time range of the measurements. The temporal window of the measurement is determined by the optical cycle of the driving lasers. This deficiency can be addressed by X-ray transient absorption.

Charge migration in C_2_HCl^+^, C_2_HBr^+^, and C_2_HI^+^ can be investigated using HHG-based attosecond water window X-rays. The scheme is shown in Fig. 3c. Like in the HHG spectroscopy experiments, the electronic superposition state can be formed by ionization of the molecules with a strong IR laser pulse. However, here the hole motion will be monitored by an X-ray probe using the transient-absorption method. Thanks to the broad X-ray supercontinuum from the HHG source, absorptions at the C K-edge and the I N-edge, Br M-edge, or Cl L-edge can be measured simultaneously. Since the adjacent atoms of the two carbons are different, the absorption due to the core-to-valence transition should also be different. The motion of the hole from one carbon to another carbon, and then to the iodine or chlorine will be monitored by the temporal variation of these absorptions. The temporal window in the X-ray transient-absorption measurements can easily cover 100 fs or longer^65^. Therefore, the femtosecond decoherence process may be observed as well.

The Born–Oppenheimer approximation is commonly used in quantum chemistry to separate fast electronic dynamics from slow nuclear motions. However, it breaks down at conical intersections where two or more potential energy surfaces are degenerate. Consequently, the coupling between electronic and nuclear motion must be considered in simulating the processes. Understanding charge dynamics around the conical intersections is important for studying photosynthesis and other photon-initiated processes. Although energy surface crossings are ubiquitous in photochemistry^80^, material science^81^, and biology^82^, direct observation of non-adiabatic dynamics at conical intersections with water window X-rays remains experimentally challenging. Neville et al. reported numerical simulations that show attosecond transient absorption at the carbon K-edge is a powerful tool to study wave-package dynamics at a conical intersection of C_2_H_2_ molecules^83^. Experimental validation of theoretical predictions of such simple molecules will pave the way to the investigation of more complex biological systems.

### Probing charge transfer in organic photovoltaic materials

**Fig. 4:**
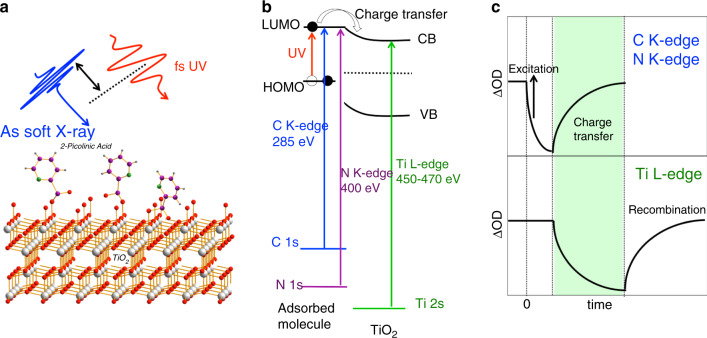
Charge transfer in organic photovoltaic materials. **a** Schematics describing 2-picolinic acid adsorbed onto TiO_2_ with femtosecond UV as the pump and attosecond SXR as the probe. **b** Energy diagram describing the electron transfer from LUMO of adsorbate to conduction band of TiO_2_. Schematics showing transitions from C K-edge and N K-edge to unoccupied states for adsorbate and those from Ti L-edge to conduction band for TiO_2_. **c** Expected attosecond transient-absorption signals at various probe energies (C K-edge, N K-edge, and Ti L-edge).

Organic photovoltaic materials have received extensive research attention due to low fabrication cost, large area production, light weight, and flexibility. One type of organic solar cell consists of two different organic molecules, namely donor and acceptor, that are mixed on the molecular level in a bulk-heterojunction structure. The most well-studied materials are poly(3-hexylthiophene-2,5-diyl) (P3HT) as a donor, and [6,6]-phenyl-C_61_-butyric acid methyl ester (PC_61_BM) as an acceptor. The electron transfer from donor to acceptor needs to be fast to compete with lossy processes, such as radiative and nonradiative recombination. Another important photovoltaic device is the dye-sensitized solar cell in which organic dyes such as N3, N719, and 2-picolinic acid are adsorbed onto the electron transport layer such as TiO_2_ to enhance the charge transfer^84,85^, as illustrated in Fig. 4a. In a strongly coupled system, the charge injection from the organic dye molecules to TiO_2_ takes place in 3–30 fs^86,87^, exceeding the instrument limit of femtosecond lasers that are the primary tool to time-resolve these processes at present^88,89^. Attosecond pulses are poised to become a powerful technique to better understand such ultrafast dynamic processes involved in improving the efficiency and functionality of solar cell materials and devices.

Attosecond X-ray absorption spectroscopy can probe resonant transitions between core electrons and unoccupied states in the valence shells of molecules, as well as the valence and conduction band of semiconductors. By utilizing a visible/UV pump with an attosecond soft X-ray probe, the electron transfer on timescales commensurate with electron and nuclei motion can be investigated. For molecules adsorbed onto TiO_2_, a few-fs UV or visible pulse can be used as the pump. During the excitation, the absorption of LUMO at the C or N K-edges (~285 eV or ~400 eV) should decrease. When the electrons move to the TiO_2_, the absorption in the conduction band probed by the Ti L-edge (~460 eV) should decrease. At the same time, the absorption of the LUMO at the C or N K-edges should increase, as shown in Fig. 4. The attosecond X-ray transient absorption at the C, N, and Ti edges could be a unique tool to measure the electron-transfer time of the strongly coupled system. The spatial localization of the 1s shell of C and N or the 2p shell of Ti means that excitation of electrons from these shells by attosecond X-ray pulses to valence electronic states provides an atomic-site-specific probe of transient valence electron/hole populations. Similar pump–probe scheme can be applied to study charge transfer between donors and acceptors in organic bulk-heterojunction solar cells.

### Controlling dielectric properties of solids

The electronic properties of matter can be modified drastically from its equilibrium state by intense laser pulses. In 2011, a substantial redshift on the band-edge (>10% of the bandgap) was reported in bulk ZnO crystals subjected to intense mid-infrared laser fields^90^. The shift was measured using a broadband ultraviolet light source in a pump–probe transient-absorption setting. In other independent studies, attosecond XUV pulses were used to probe the dynamical absorption, on the subcycle timescale of the driving laser field, using thin Si^91^ as well as diamond^92^. Reversible semi-metallization of insulating materials has been demonstrated in 2013, when SiO_2_ was illuminated with intense few-cycle near-infrared Ti:Sapphire-laser pulses^93^. A semi-metallization model was proposed to explain the transient-absorption experiments at the Si L-edge (100–110 eV) that was covered by the spectral range of the Ti:Sapphire-laser-driven attosecond pulses. The attosecond water window X-ray source will allow simultaneous measurements at both the Si L-edge and the O K-edge (533 eV) in transient-absorption experiments. This could provide the evidence of correlated charge motion at the Si and O sites to deepen the understanding of the physical mechanism. Other materials such as diamond, BN, and Si_3_N_4_ can also be investigated in the similar manner. Controlling electrical conductivity of solids at the timescale of an optical cycle allows a way to extend the speed of optoelectronics into the petahertz (10^15^ Hz) domain^94^, far exceeding the limit of current field-effect transistor-based semiconductor electronics. This is critical to applications of high-speed all-optical signal processing and to the development of optical-field-effect devices^95,96^.

## High-harmonic spectroscopy of condensed matter

High-harmonic spectroscopy has been widely used in atomic and molecular systems^97^, but that knowledge cannot be directly implemented in condensed matter systems because underlying microscopic dynamics are different. In solid-state materials, the laser field-driven electrons are in the proximity of the periodic Coulomb potential, so the usual strong-field approximation^98^, which is the foundation of the three-step re-collision model^4,5^, becomes qualitatively invalid.

**Fig. 5:**
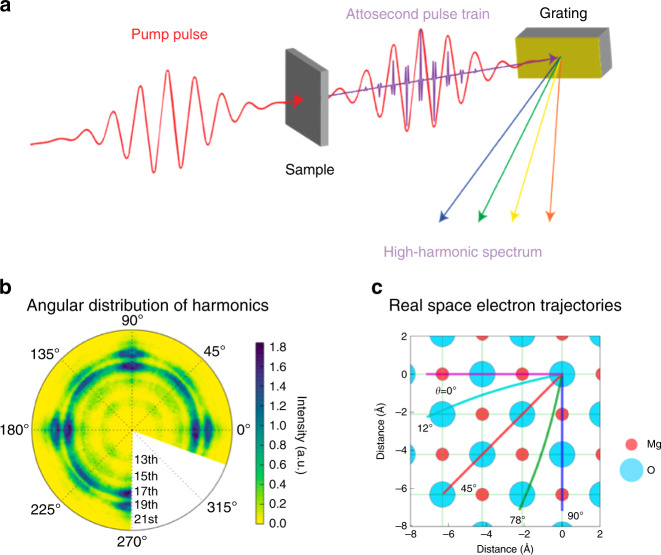
High-harmonic spectroscopy of solid materials. **a** Schematics for HHS of solid-state materials in transmission geometry. Few-cycle laser pulses excite the sample at the V/Å level without damage. Attosecond pulses are produced on subcycle timescales, which interfere in the far field and produce discrete peaks in high-harmonic spectrum. **b** Measured crystal orientation-dependent high-harmonic spectrum from wide-bandgap cubic MgO crystal. **c** Real-space electron trajectory model showing how harmonic signal increases (decreases) when the electron trajectories connect (miss) neighboring atomic sites. If trajectories connect the first nearest neighbors (O–Mg), they produce the strongest signal, as seen along cubic directions. If they connect the second nearest-neighbor (O–O) secondary maxima are produced, as seen along diagonal direction of the cube. Reproduced with permission from ref. ^15^, Copyright 2017, Springer Nature.

Recently, a real-space electron trajectory model has been developed that considers the role of the periodic potential on the semiclassical motion of the electron and includes the possibility of coherent collisions with the neighboring atomic sites^15,19^. In this picture, harmonic emission becomes stronger (weaker) when electron trajectories strike (miss) the atomic cores of nearest-neighbor atoms. A typical experimental setup for such spectroscopy is shown in Fig. 5a. In this particular example, a wide-bandgap MgO crystal is pumped with a NIR laser pulse, and XUV harmonics are analyzed as a function of crystal orientation and laser ellipticity. The real-space electron trajectory model describes the observed crystal orientation dependence, ellipticity dependence, and therefore provides the opportunity to probe the valence charge density distributions inside bulk materials in all-optical settings^15,19^. Because the electron trajectories can be controlled by laser parameters (field strength, polarization, and wavelength), the real-space picture provides a powerful, all-optical, and tabletop approach to probe valence charge density distributions in bulk materials.

**Fig. 6:**
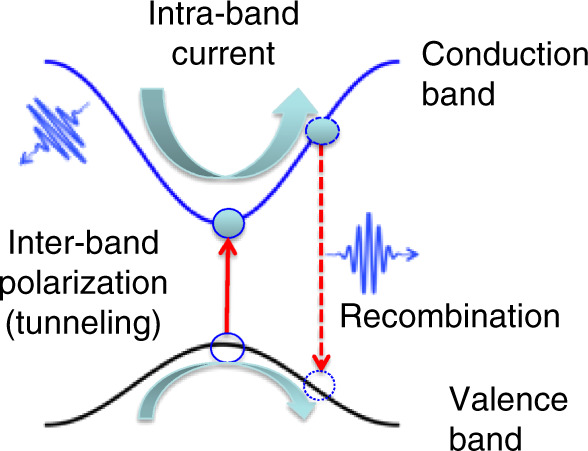
Momentum-space interpretation for HHG in solid materials. The microscopic mechanism for solid-state HHG showing two major channels semiclassically. The laser field promotes an electron from valence to conduction band leaving behind a hole in the valence band, as shown by the vertical solid line. Both the electron and hole are accelerated in their respective bands. Channel 1: the nonlinear intraband current in the conduction band can radiate. Channel 2: the electron can recombine with the hole at a later time, as shown by the dashed line, and emit a high-energy photon. Adapted with permission from ref. ^14^, Copyright 2019, Springer Nature.

Dynamics in solid-state materials are often described in reciprocal or momentum space. In this framework, non-perturbative high-order harmonics originate mainly from two channels, namely the intraband nonlinear current and the interband polarization, as shown schematically represented by simple cosine bands of opposite mass across a direct bandgap in Fig. 6^14^. Initially, the valence band is full, and the conduction band is empty. Upon photoexcitation, an electron–hole pair is created at the zone center (*k* = 0) by tunneling across the minimum bandgap. Then, both the electron and the hole are accelerated by the electric field towards the zone edge. At high-enough peak-field strength, the electrons can reach the zone edge (*k* = π/a, where a is the lattice constant) and be Bragg diffracted, even multiple times every half-cycle. For nonparabolic bands such intraband current can radiate at much higher frequencies than the fundamental driving frequency. Similarly, the electron and hole can recombine at later time at higher crystal momentum locations, as shown by the dashed arrow, releasing a high-energy photon^99,100^. Often at high peak fields these dynamics get coupled.

Through the intraband channel, the dispersion of a responsible conduction band can be probed, as proposed by Ghimire et al. ^12^ and implemented in detail by Luu et al. using quartz crystal recently^101^. In contrast, the interband channel involves intrinsic atto-chirp because there are unique recombination paths at different energies and momenta corresponding to different harmonic orders^99,100^. Therefore, by measuring the atto-chirp of high harmonics from the interband channel, the momentum-dependent bandgap can be deduced, as shown in ZnO crystal^20^. At sufficient high-harmonic peak intensities, transitions to higher-lying conduction bands is possible and that could lead to secondary plateau in high-harmonic spectrum, as observed in rare-gas solids^16^ and in room temperature solids, such as MgO and SiO_2_ crystal^102^. Similarly, depending on the system, lower-lying valence bands may also contribute, as understood by analysing the terahertz-field driven harmonics in GaSe crystal^103,104^. Therefore, in the momentum-space framework, high-harmonic spectroscopy also presents a novel, all-optical approach for probing electronic band structure, including away from the zone center. For these measurements it may be essential to separate propagation effects.

### Dipole phase and propagation effects

**Fig. 7:**
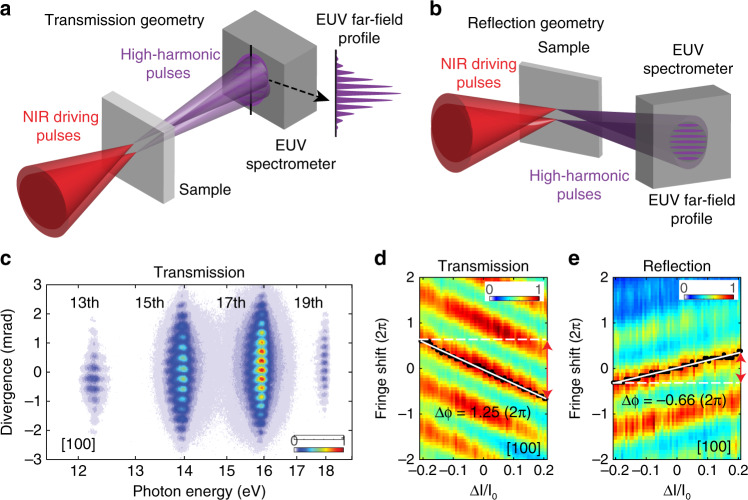
Attosecond interferometry in transmission and reflection configuration. Experimental setup in **a** transmission and **b** reflection, where high harmonics from two sources interfere in the far field. Reproduced with permission from ref. ^106^, Copyright 2019, Springer Nature. A representative interferogram is shown in **c**. Fringe shift as a function of relative change in laser intensities, in transmission. **d** and in reflection (**e**). Reflection geometry closely represents the microscopic effects. Fringe shifts in transmission are overwhelmed by propagation effects. Adapted with permission from ref. ^106^, Copyright 2019, Springer Nature.

One of the unique challenges in probing atto-chirp, and for HHS in general, is separating the strong propagation effects. This is because in bulk samples, the intense pump laser pulse accumulates a significant nonlinear phase as it propagates from the entrance to the exit end from where the above bandgap harmonics are typically emitted from. Modeling the effects of the nonlinear phase, such as the effects from $\chi ^{\left( 3 \right)}$ on to the fundamental laser field and consequently to high-order harmonics, is complex. Recently, non-perturbative high harmonics were also observed in the backward reflection geometry, which avoids propagation effects^105^. In the following passage, we briefly discuss the results of attosecond interferometry in a homodyne configuration that was performed in both a reflection and a transmission geometry^106^. The experimental setup is shown in Fig. 7. Here, the main idea is that XUV harmonics are generated in two spatially separated foci, but harmonics overlap in the far field and self-referenced interferometry is performed. The measured fringe shifts in the reflected harmonics were reproduced by separating the interband polarization in a strongly driven two-level system. The fringe shifts in transmission are overwhelmed by propagation effects, as  the shifts are much larger than the predictions from microscopic model and are opposite in direction.

Intriguingly, the measured fringe shifts in reflection mode are source material-dependent, as XUV harmonics from MgO crystal show much larger shifts compared with those from SiO_2_. This is in contrast to the gas-phase HHG where, for high-enough photon energies, the dipole phase is independent of the target atom^107^. While it is not immediately clear what specific features of the electronic structure make the dipole phase shift differently in these materials, the experimental results clearly show the sensitivity of high-harmonic spectroscopy. Along this line, future experiments can utilize this platform in many ways; for example, by referencing novel high-harmonics sources such as from solids, nanostructures, or liquid media with respect to that from well-studied gas-phase sources in a heterodyne configuration. HHS could also be used to probe impurities in semiconductors with high spatial resolution.

### Compact XUV light source based on solid-state HHG

Recent results have shown that solid-state HHG has the following main advantages when compared with HHG from dilute gas targets: (i) lower prerequisite on driving peak intensity because solid-state HHG threshold on dielectrics is around 10^12^ W cm^−2^^16^, (ii) the XUV waveform produced by some solid-state materials are found largely immune to fluctuations in the driving fields^18^, and (iii) the use of tailored nanostructures, such as metal–sapphire bow tie^108^, or the use of heterostructures, such as epsilon-near-zero materials, improves the HHG efficiency substantially^109^. The use of zone plates as a source material has provided the ability to focus the high-harmonic beams directly without the use of additional optics^110^ that could be extremely demanding in the XUV wavelength range. Solid-state HHG setups do not require sophisticated high-vacuum pumping systems because of no gas load. Polarization of solid-state harmonics can be controlled by taking advantage of anisotropy in the electronic band structure^111^ as well as the Berry phase in non-centrosymmetric crystals, such as SiO_2_^112^. Circularly polarized harmonics have been demonstrated, at least in the visible wavelength range^113^.

One drawback of solid-state HHG when compared with gas-phase HHG is that the high-energy cutoff is limited by the material’s damage threshold, which in the case of wide-bandgap dielectric, has reached a ceiling of ~40 eV^16^. Further extension to higher photon energy range might be possible by the use of ultra-short driving pulses. The rapid development of few-cycle, high-repetition-rate light sources, including optical parametric chirped-pulse amplifiers, can provide suitable pump sources for solid-state HHG. With field enhancement in nanostructures in wide-gap materials^108^, high-harmonic XUV light sources based on laser oscillators or OPCPAs operating at high-repetition rates appear promising. The dispersion of solids in the infrared pump wavelength range can be engineered, for example, by introducing waveguides^114^, photonic crystal structures^115^ and metamaterials^116^, and by utilizing epsilon-near-zero effects through doping^109^. The phase matching between pump fields and harmonics in a specific spectral range could be optimized, which could lead to enhanced generation efficiency. In the high photon energy regime, the absorption of harmonics above a material’s bandgap reduces the effective generation thickness to the absorption length—usually on the order of ten nanometers for XUV harmonics—thus greatly limiting the HHG efficiency. For sufficiently high photon energy ranges, the absorption of high-energy photons could be alleviated. For example, for just under 100 eV, silicon’s attenuation length is expected to be around 500 nm. If phase matching can also be achieved, for example by taking advantage of a tunable refractive index in epsilon-near-zero materials, solid-state HHG could also reach the SXR photon energy range.

### All-optical probing of ultrafast dynamics in materials

There are initiatives aimed at testing the potential of the HHG process in probing structure and ultrafast dynamics in condensed matter systems. Examples of ultrafast phenomena include insulator to metal phase transitions, strongly correlated electron dynamics^117^, and topological phase transitions in quantum materials^118^. Theoretical predictions are emerging, which includes calculations on one dimensional model systems focusing on how the trivial to topological phase transition dramatically enhances the efficiency of the HHG process^118^ and two-dimensional Haldane model system focusing on how HHG could probe topological invariants^119,120^. This particular aspect, as well as just how phase transitions and correlations manipulate the HHG process at the microscopic level on novel quantum materials, are outstanding questions at this time^121^. A nominal experimental setup could be a pump–probe scheme, wherein an ultrafast pump laser initiates a phase transition, and a strong-field probe pulse produces high harmonics from the active medium as a function of pump–probe.

Currently, angle-resolved photoemission spectroscopy (ARPES) is the standard spectroscopic method to measure the band structure. However, since it is based on the detection of electrons, it has stringent experimental requirements such as ultra-high-vacuum and ultra-clean sample environments. Its temporal resolution in a pump–probe setting has been demonstrated only down to about tens of femtoseconds^122^. Similarly, time-resolved X-ray absorption/diffraction techniques also have limitations, usually >50 fs, because of the temporal jitter between the pump laser and the X-ray source^123^. Being an all-optical method, high-harmonic spectroscopy provides unprecedented time resolution and also much of these measurements can be performed in ambient conditions, i.e., without the need for vacuum apparatus, especially if the pump lasers are in the mid-infrared wavelength range. High harmonics provide a novel approach to probe active electronics in two-dimensional materials and heterostructures.

Finally, we note that so far the solid-state HHG theory is largely based on single active electron approximation, either through solving time-dependent Schrödinger equations (TDSE)^99,100,124^ or through semiconductor Bloch equations (SBE)^103,125^. Many-body effects such as electron-electron correlations and dephasing are largely ignored while the latter is implemented just phenomenologically. Therefore, more predictive theories that consider correlations and multiple electronic bands are highly desired. One path forward could be through the ab initio approach based on time-dependent density-functional theory (TDFT), which could in principle consider the full band structure and joint density of states^126^.

## Summary and outlook

The recent breakthroughs in attosecond “water window” X-ray sources open the door to game-changing applications. The combination of unprecedented time resolution with element-specific spectroscopy provides a unique opportunity for studying charge and nuclear dynamics in organic molecules and materials containing C, N, O, and other important elements and may help in understanding the charge transfer mechanism in solar cell materials. So far, transient-absorption spectroscopy measurements have been demonstrated in the water window with attosecond time resolution. It is expected that attosecond photoelectron spectroscopy^127^, attosecond coherent diffraction imaging^128^, attosecond reflection spectroscopy^129^, as well as other time-resolved techniques^130^, such as HHG-based ARPES^131^ and Cold Target Recoil Ion Momentum Spectroscopy (COLTRIMS) apparatus^132^ will be implemented beyond the Carbon K-edge.

Currently, the attosecond pulse energy from HHG source is too weak to populate excited state in the atoms or molecules. The benefit of more energetic attosecond pulses is the possibility to perform true attosecond-pump–attosecond-probe experiments, where excitations are fulfilled through direct core level transition by absorbing a single XUV/SXR photon. Major efforts towards enhancing HHG efficiency include two-color driving fields as well as the use of MWIR. In the latter, it is easier to individually control the three steps of the HHG process^133^ because of the long laser cycles. Recently, a fundamentally different technique for producing attosecond pulses has emerged at X-ray free-electron lasers, particularly at the Linac Coherent Light Source (LCLS)^134,135^. Accelerator-based sources typically provide substantial flux so they will enable a new class of experiments, including true attosecond-pump and attosecond-probe capabilities.

Solid-state HHG has been realized in a wide range of materials and harmonic spectrum has reached ~40 eV. These results have shown promises for stable attosecond pulses in compact experimental setups, along with the novel all-optical method to directly probe the structure and dynamics of the source material. Because of the modest requirements in the peak intensity (10^11^–10^13^ W cm^−2^) solid-state HHG could possibly be pumped with next-generation high-repetition rate fiber laser systems such that the total flux of XUV photons can be increased significantly. The underlying microscopic generation mechanism can be utilized to probe both structure and dynamics of the source materials. When we consider the fact that the driven electron motion in solids must be governed by their band structure, high harmonics bear the imprint of the electronic band structure of the solid. A unique capability of HHG approach is the ability to access conduction bands lying higher than the Fermi level, and yet the polarization response and spectral phase of harmonics provides additional sensitivity such Berry curvature and topology, which are not directly accessible with conventional spectroscopic methods such as ARPES. While the community is still in the process of developing the detailed decoding procedure, high-harmonic spectroscopy has quickly become an attractive area of attosecond science and technology.

With the emergence of new generation tabletop attosecond light sources based on both gas-phase and solid-phase HHG and novel time-resolved experimental and theoretical tools in a wide spectral range for studying dynamics in isolated molecules and condensed-phase materials, we now stand at the threshold of another revolution in attosecond science and technology.
